# Supporting evidence-informed policy and scrutiny: A consultation of UK research professionals

**DOI:** 10.1371/journal.pone.0214136

**Published:** 2019-03-26

**Authors:** Lindsay A. Walker, Natalia S. Lawrence, Chris D. Chambers, Marsha Wood, Julie Barnett, Hannah Durrant, Lindsey Pike, Gerard O’Grady, Sven Bestmann, Andrew P. Kythreotis

**Affiliations:** 1 Cardiff University, Cardiff, United Kingdom; 2 University of Exeter, Exeter, United Kingdom; 3 IPR Bath, University of Bath, Bath, United Kingdom; 4 PolicyBristol, University of Bristol, Bristol, United Kingdom; 5 University College London, London, United Kingdom; 6 University of Lincoln, Lincoln, United Kingdom; Mercy Hospital, SIERRA LEONE

## Abstract

Access to reliable and timely information ensures that decision-makers can operate effectively. The motivations and challenges of parliamentarians and policy-makers in accessing evidence have been well documented in the policy literature. However, there has been little focus on research-providers. Understanding both the demand- and the supply-side of research engagement is imperative to enhancing impactful interactions. Here, we examine the broader experiences, motivations and challenges of UK-based research professionals engaging with research-users relevant to policy-making and scrutiny in the UK using a nationwide online questionnaire. The context of the survey partly involved contributing to the UK Evidence Information Service (EIS), a proposed rapid match-making service to facilitate interaction between parliamentary arenas that use evidence and research-providers. Our findings reveal, at least for this sub-sample who responded, that there are gender-related differences in policy-related experience, motivations, incentives and challenges for research professionals to contribute to evidence-informed decision-making through initiatives such as the EIS. Male and female participants were equally likely to have policy experience; however, males reported both significantly broader engagement with the research-users included in the survey and significantly higher levels of engagement with each research-user. Reported incentives for engagement included understanding what the evidence will be used for, guidance on style and content of contribution, and acknowledgement of contributions by the policymaker or elected official. Female participants were significantly more likely to select the guidance-related options. The main reported barrier was workload. We discuss how academia-policy engagement initiatives can best address these issues in ways that enhance the integration of research evidence with policy and practice across the UK.

## Introduction

Decision-makers operate effectively when they have timely access to reliable information. The concept ‘evidence-informed policy’ initially gained recognition in the UK with the Labour Government from 1997 (e.g. [1]) and was linked to pressures for improved effectiveness and accountability [2]. Since then, the idea has broadened to incorporate a more complex understanding of how policy and evidence relate (see [2,3]). More recent debates have focussed on research engagement to facilitate evidence-informed decision-making. For example, UK government departments now release details outlining their areas of research interest (ARIs) with the aim to ‘*align scientific and research evidence from academia with policy development and decision-making*’ [4]. This was in response to Sir Paul Nurse’s recommendation while reviewing the UK Research Councils to ‘*secure greater engagement*’ between policy-makers and researchers [5]. Additionally, the well-established UK Research Excellence Framework (REF) and the recently announced UK Knowledge Exchange Framework (KEF) provide a financial incentive for universities to demonstrate the impact of research findings [6,7]. Indeed, evidence suggests that the REF has contributed to a culture of wider engagement [8]. However, a recent and substantial study examining the role of research in the UK Parliament revealed that although research in its broadest sense is useful for parliamentary work, academic research is ‘*not cutting through*’ [9]. The study revealed that challenges to the use of academic research included lack of accessibility and poor communication [9], which mirrored findings from an earlier study of eight UK parliamentary staff [10]. Yet there remains a gap in knowledge regarding the challenges and motivations for research professionals to engage with evidence-informed policy-making processes. Understanding both the demand- and the supply-side of academic-policy engagement is essential for enhancing impactful interactions.

Academic research is only one source of evidence used by the UK Parliament—parliaments are interested in knowledge of all types [9]. Different parts of parliament use and access evidence in diverging ways depending on their function [10]. Geddes et al. (2017) revealed that recognising these differences in evidence use, as well as understanding how Parliament works, is crucial for academics to effectively engage. This suggests that academics are required to provide different types of knowledge to the different parliamentary arenas that use evidence. Although this might indeed be a barrier to academic engagement, to date no study has surveyed the motivations and barriers for research professionals to engage with processes relating to policy-making and scrutiny.

Alongside the focus on research engagement there has been a rise in the number of knowledge mobiliser roles [11], for example knowledge brokers within universities [12] and Knowledge Exchange Fellows (e.g. [13]). These roles aim to translate knowledge into action to address the identified ‘gap’ between research and decision-makers [14–16]. Indeed, a large body of academic research has focussed on solutions to bridging this gap (e.g. [17–21]). However, much of this work has focussed on processes involving the Executive, with Parliament’s policy influence only recently gaining increased recognition [22–24].

Even with this increased attention, challenges around using academic research in a parliamentary context remain. For example, the higher education sector is typically underrepresented in evidence submissions to Select Committees and Public Bill Committees within the UK Parliament [9]. In part to address this, the Parliamentary Office for Science and Technology (POST) has developed an online hub for academic researchers to access guidance and information on providing evidence to parliamentary arenas. This is alongside the popular *Research*, *Impact and the UK Parliament* training workshops delivered at university campuses across the UK by the Universities Programme at the Houses of Parliament and POST [25]. Further studies on the supply-side of research engagement has been identified as ‘*instructive*’ by POST [9], indicating that exploring the academia side of engagement would be useful and informative. The devolved legislatures are also all developing their own academic engagement programmes.

This paper contributes to the researcher engagement debate by improving our understanding of the policy experience of UK-based researchers. Our research was conducted as part of a proposed initiative called the UK Evidence Information Service (EIS), an innovative model that aims to facilitate research engagement with decision-makers including parliamentarians [26]. The proposed EIS aims to act as a rapid matchmaker to connect the UK research community and decision-makers in the service of scrutiny and development of evidence-informed public policy. By matchmaking evidence-users with evidence-providers, the EIS should improve the communication and accessibility of research findings, addressing a key barrier to current evidence-informed decision-making processes [27,28]. A previous consultation of 56 UK parliamentarians revealed that 85% supported the EIS [26]. This consultation led to the EIS working with in-house parliamentary research services to explore potential pathways for information flow from the academic community to parliamentary arenas via supporting existing services. The EIS would complement and enhance the parliamentary research services that parliamentarians and their staff already utilise by providing rapid access to a wide network of academics. It is also proposed that as part of the EIS, contributing academics would be trained and briefed in parliamentary engagement.

In this paper we explore the broader experiences and motivations of an opportunistic sub-sample of UK-based researchers engaging with research users in the UK using a nationwide online questionnaire. This survey of the ‘supply-side’ of academic-policy engagement complements our ‘demand-side’ research comprising the previous consultation of UK Parliamentarians [26] and ongoing discussions with participating parliamentary research services. We also investigate what could encourage or pose a challenge to researchers engaging with academic-policy initiatives such as the EIS. We then examine how academic-policy initiatives such as the EIS might operate so to enhance research engagement. Although part of the survey questions were asked in the context of a proposed EIS system, the findings have a wider application to academic-policy engagement initiatives. For example, the results suggest features that would encourage academic participation if incorporated into an academic-policy framework.

## Methods

### Online questionnaire

Data were collected via an anonymous online questionnaire between 26 March 2017 and 25 January 2018. The survey was designed by the authors with input from the House of Commons Library and the National Assembly for Wales Research Service. Ethics approval for the survey was awarded by the School of Geography and Planning at Cardiff University on 06 March 2017. The survey was chosen to be distributed online as a cost-effective method of data gathering. The questions aimed to gather information on the prior experience of academics contributing to material that politicians could use, including the nature of these interactions and the motivations to engage. The survey also collected data on future contributions, such as what would encourage or pose a challenge for academics to provide evidence as well as the logistics of an academic-policy framework. The full survey of 22 questions is included in S1 Table. The survey was generated using Qualtrics software [29].

The survey was circulated to 72 Universities / Research Institutes based in the UK. This was achieved through both publicly available email addresses (e.g. through Head of College / Faculty) and the university contact lists of the four UK legislative bodies (e.g. through Knowledge Exchange Officers). This ensured that the survey was circulated across the UK. Seventy-four (74) UK learned societies, one charity and one network (the latter two focussing on equality and diversity in higher education) were also sent the survey with a request to circulate to their members. The survey was also promoted on social media. It was requested that only UK-based academics and researchers complete the survey.

### Categorising participants

Career stage information provided by participants (see Supplementary Information for full list of options) was categorised by the authors into the three groups ‘early-’, ‘mid-’ and ‘senior-career’ and defined as follows. The category ‘early’ included those participants who identified as; PhD researcher, Research technician, Research assistant, MSc researcher. The category ‘mid’ included those participants who identified as; Lecturer, Research Fellow, Teaching Fellow, Post-doc position. The category ‘senior’ included those participants who identified as; Professor, Associate Professor, Senior Lecturer or Reader. The authors note that analyses involving career stage categorisations are indicative rather than conclusive as the distinction between roles can vary within and between universities. Inclusion of career stage in relevant models is explained in full below.

Academic discipline information provided by participants was categorised by authors using the Joint Academic Coding System (JACS) 3.0 principle subject codes. JACS is used and maintained by the Higher Education Statistics Agency (HESA) and the Universities and Colleges Admissions Service (UCAS) to classify academic subjects, with JACS 3.0 utilised since 2012/13. Using these JACS 3.0 codes, the responses were further categorised into ‘social sciences/art and humanities’ and ‘natural sciences’. Five responses were not possible to classify using JACS 3.0 and therefore were not included in any related analysis. Inclusion of science discipline in relevant models is explained in full below.

We were interested in exploring the impact of reported demographic factors on the survey results. As we could reliably analyse associations with gender due to the sample size (see Results for full demographic breakdown of respondents), we concentrated our analyses on gender where possible to add to the understanding of any gender-specific motivations and/or challenges to engage. We analysed for other demographic effects (e.g. career stage) where the sample size allowed.

## Analysis

All statistical analyses were conducted with R v 3.3.0 [30]. For brevity, the questions asked of the data and the accompanying analyses are outlined in full in Table 1. Where possible, generalized linear models (as indicated by ‘GLM’) or generalized linear mixed effects model (as indicated by ‘GLMM’) were used. In these occasions, all models were checked for normality of residuals and homogeneity of variance. Statistical modelling utilized a stepwise model simplification approach. All fixed terms were fitted together and then the non-significant terms with the least explanatory power were sequentially removed. This occurred until a minimal adequate model was reached, retaining only those predictors whose removal now yielded a significant reduction in the explanatory power of the model. For GLMM, all random terms were retained in the model throughout. Statistical significance is reported from the calculations in the change in explanatory power on removal of that term from the model. Each model is explained in full below.

**Table 1:** Questions for analysis and the statistical methods applied.

Question for analysis	Statistical model/test applied	Response variable	Explanatory variable(s), if applicable
*Policy experience of a sub-sample of UK-based research professionals*			
The impact of gender on a participant reporting or not reporting a previous engagement with a source of material containing evidence that politicians could use (see S1 Table for list of options included in survey).	GLM with a binomial error distribution	Reporting or not reporting (yes, no) a previous engagement	Fixed effect: gender
If gender influenced the number of reported non-zero previous engagements with a source of material containing evidence that politicians could use.	GLM with a binomial error distribution	Number of reported non-zero previous engagements (fixed maximum of 12 possible options)	Fixed effect: gender
Association of gender with the number of reported previous engagements with a source of material containing evidence that politicians could use while controlling for the variation in reported engagement between the different sources.*	GLMM with a binomial error distribution	Number of reported previous engagements (excluding ‘not heard of’ responses)	Fixed effect: gender Random effect: source of material
Association of gender with the nature of previous interactions.	Two-sample proportion test for equality without continuity correction	Number of participants who did and did not select each option	
Association of gender with the motivations for previous interactions.	Two-sample proportion test for equality without continuity correction	Number of participants who did and did not select each option	
If reporting the provision of specific or general advice was related to gender.	Chi-square test of independence	Number of participants in each category	
*Future provision of evidence by a sub-sample of UK-based research professionals*			
If gender or having previous policy experience influenced whether participants reported that they would like to take part in academic-policy initiatives such as the EIS.**	GLM with a binomial error distribution	Indication of involvement (yes, maybe) with future academic-policy initiative	Fixed effects: reported previous policy experience (yes, no), gender
If science discipline influenced whether participants reported that they would like to take part in academic-policy initiatives such as the EIS.**	GLM with a binomial error distribution	Indication of involvement (yes, maybe) with future academic-policy initiative	Fixed effect: science discipline (social sciences/arts and humanities, natural sciences)
Association of (i) gender, (ii) career stage, and (iii) science discipline with what would encourage contributions to academic-policy initiatives	Two-sample proportion test for equality without continuity correction applied to (i)-(iii)	Number of participants who did and did not select each option	
Association of (i) gender, (ii) career stage, and (iii) science discipline with what would pose as challenges to academic-policy initiatives	Two-sample proportion test for equality without continuity correction applied to (i)-(iii)	Number of participants who did and did not select each option	
*Format of academic-policy initiatives*			
If gender was independent of the reported importance of having contributions to academic-policy initiatives such as the EIS recognised	Chi-square test of independence	Number of participants in each category	

* Participants were asked whether they had ‘contributed to’, been ‘approached by, but not contributed to’, ‘heard of only’ or ‘not heard of’ for each of the different sources of material containing evidence that politicians could use (see S1 Table for list of options included in survey). To obtain a reliable reflection of engagement levels, individuals who selected the response ‘not heard of’ for a given option where excluded, thereby providing an engagement level for research professionals who indicated they had at least heard of the option.

** The response variable only included the responses ‘Yes’ and ‘Maybe’ as there were too few ‘No’ responses (*n* = 5) to statistically analyse.

## Results

**Fig 1:**
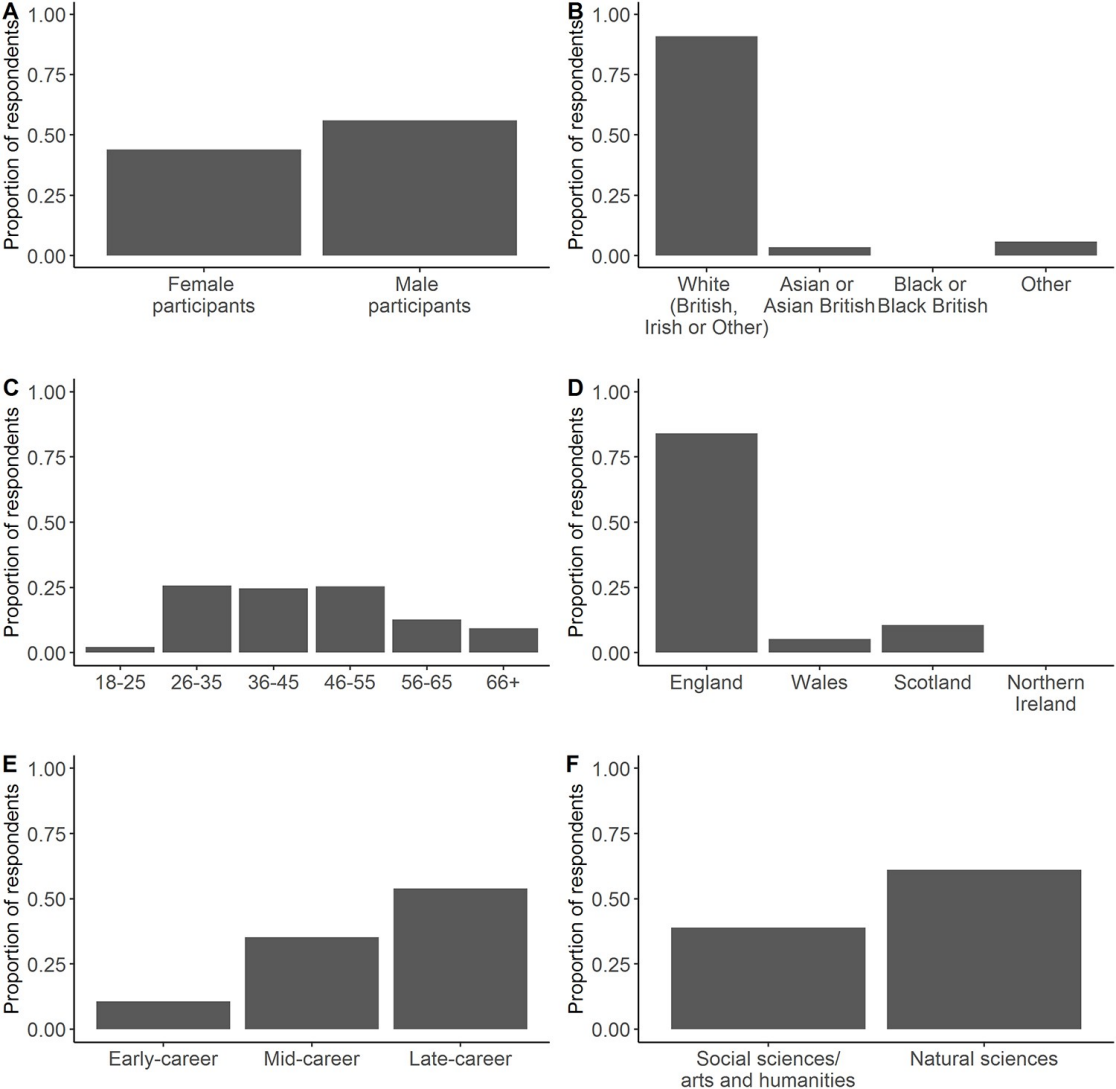
Self-reported demographic information of participants. A. Gender (*n* = 268 individual respondents). B. Ethnic group (*n* = 245 individual respondents). C. Age bracket (years; *n* = 268 individual respondents). D. Location of work (*n* = 207 individual respondents). E. Career stage (see Methods for definitions; *n* = 215 individual respondents). F. Science discipline (*n* = 262 individual responses that were classifiable by JACS 3.0).

A total of 461 respondents initiated the survey, with 132 respondents not answering a single question. Of the 329 who completed at least one question, 283 completed > 75% of the questions, with 154 completing all survey questions. See Fig 1A–1F for demographic information of survey participants. Six individuals specified a location of work place outside of the UK; however, as the invite requested that only UK-based research professionals completed the survey, it was assumed that these six individuals were based in the UK. For example, researchers can reside in different countries to their employers, be on long-term sabbatical leave, or have an honorary status with a University in a different country. Furthermore, five of the six individuals reported that they had at least heard of some of the UK policy-related options in the survey, with two individuals reporting previous engagement (see Question 2 in S1 Table for list of options). The six individuals were therefore retained in the dataset with this assumption. All sample sizes are included with the relevant analysis below. NA responses were excluded in all analyses.

All data and analyses are publicly available via the Open Science Framework [31]. As detailed in the Methods section, we analysed associations with demographic factors where the sample size allowed. The statistical methods for the following three sub-sections are detailed in Table 1.

### Policy experience of a sub-sample of UK-based research professionals

**Fig 2:**
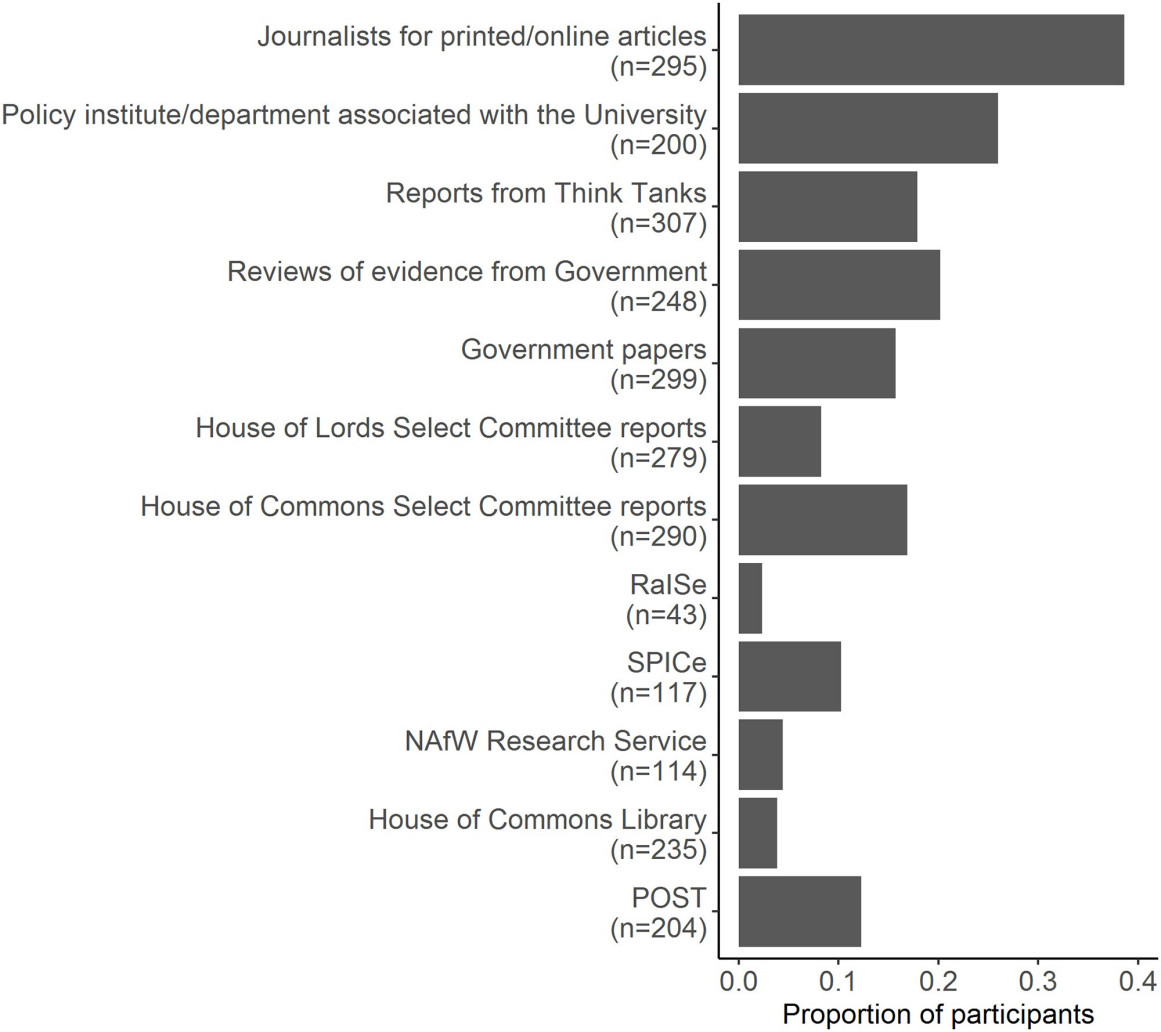
The proportion of participants that reported previous engagement that had at least heard of the option. Number of individual respondents who had at least heard of the option indicated in the y-axis. Abbreviations: RaISe, Northern Ireland Assembly Research and Information Service; SPICe, Scottish Parliament Information Centre; NAfW, National Assembly for Wales; POST, Parliamentary Office of Science and Technology.

Of 324 respondents, 314 felt that it was important for research to be considered during policy making. When asked about engaging with sources of material containing evidence that politicians could use (see Fig 2 and S1 Table for list of options included in survey), 165 (*n* = 321 respondents) reported they had engaged with at least one. There was no statistically significant association of gender with whether a participant reported or did not report a previous engagement (GLM: χ^2^_1_ = 1.03, P > 0.05; *n* = 266 individual respondents). The number of engagements with sources of material containing evidence that politicians could use ranged from zero to 12 out of a possible 12 options (median: 1). Looking at only those who reported one or more engagements, gender was statistically significant, with men reporting more engagement across the available options (GLM: χ^2^_1_ = 8.24, P < 0.01; *n* = 140 individual respondents; median: 2).

**Fig 3:**
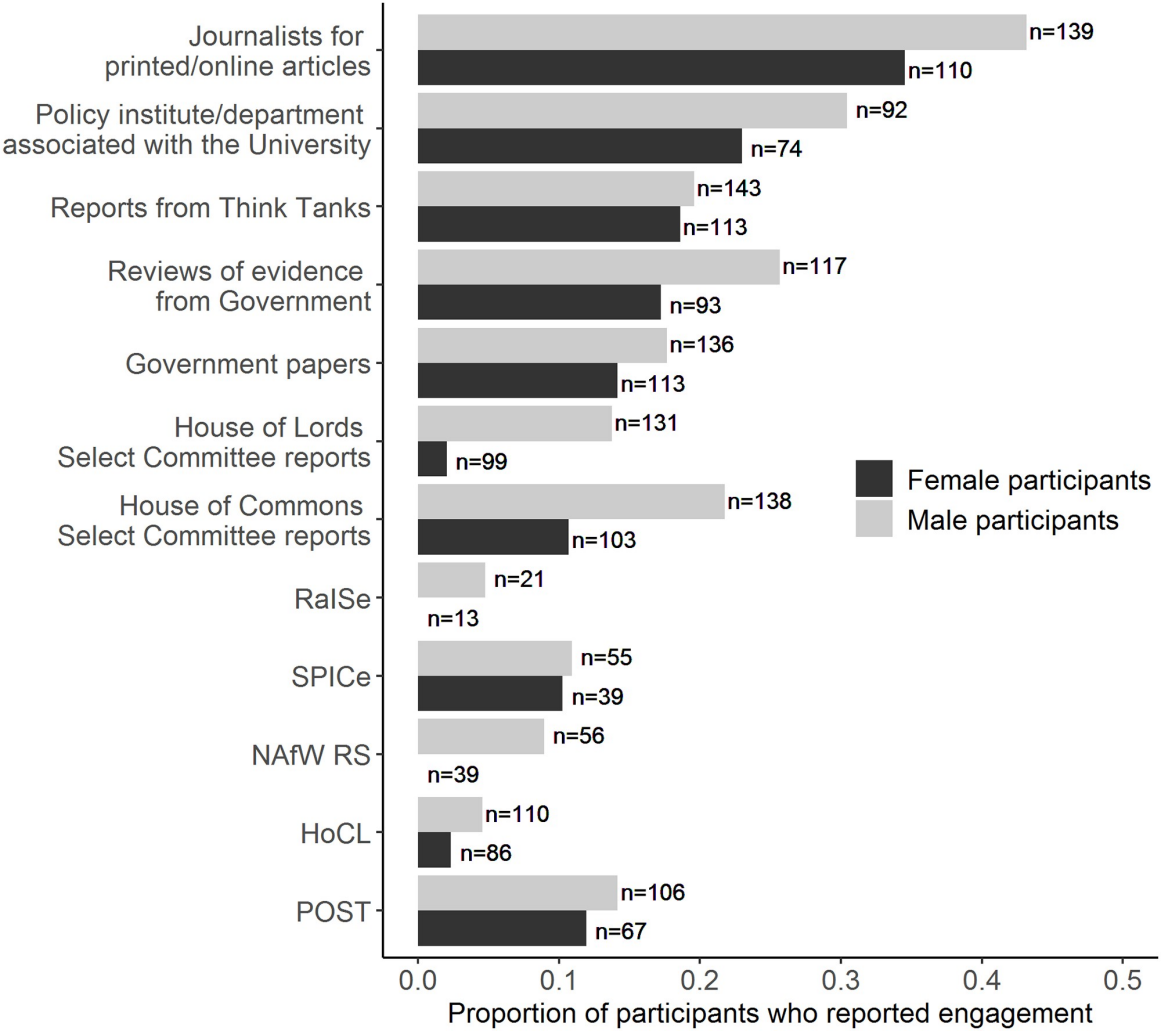
The proportion of participants that reported previous engagement that had at least heard of the option while also providing gender information. Number of individual respondents who had at least heard of the option per gender indicated on the graph. Male participants were significantly more likely to report engagement. Abbreviations: RaISe, Northern Ireland Assembly Research and Information Service; SPICe, Scottish Parliament Information Centre; NAfW, National Assembly for Wales; POST, Parliamentary Office of Science and Technology.

Respondents also reported a difference with what they had engaged with. To obtain a reliable reflection of engagement levels, only individuals who indicated they had at least heard of the option were included in the analysis (see Methods for full details). The greatest reported contributions were with printed / media content (114 of 295 individual respondents who had heard of the option), followed by the policy institute / department associated with their university (52 of 200 individual respondents who had heard of the option; see Fig 2 for full results). While controlling for the variation between the different options, it was found that men were significantly more likely to report an engagement across the different options (GLMM: χ^2^_1_ = 15.25, P < 0.001; *n* = 266 individual respondents; see Fig 3).

**Fig 4:**
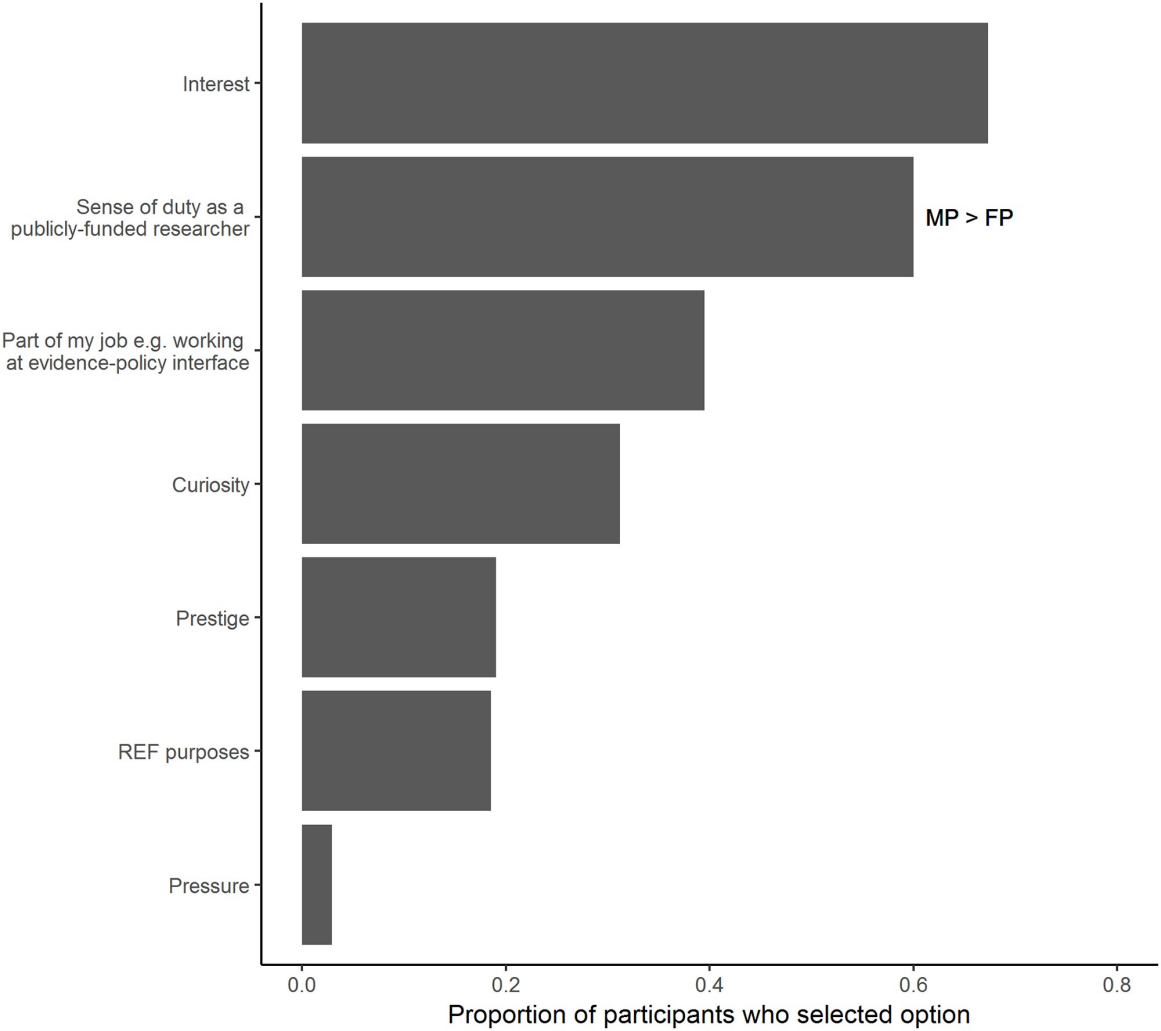
The reported motivations for providing evidence for policy-making (*n* = 205 individual respondents). Looking at those responses where gender information was also provided, male participants (MP) were significantly more likely to select sense of duty than female participants (FP) (*n* = 175 individual respondents; as indicated by MP > FP).

Engaging with these sources of material containing evidence that politicians could use was mainly to provide general advice on the research topic (*n* = 127 of 187 respondents), although this was closely followed by talking about their own research projects (*n* = 113 of 187 respondents). Twelve responses recorded under ‘other’ included several references to both government/ and parliamentary process—see S1 Appendix. Male participants were more likely to report providing general advice than female participants (χ^2^_1_ = 4.01, P < 0.05; *n* = 158 individual respondents), but equally likely in all other three options (χ^2^_1_ < 1.66, P > 0.05 for all). Contributing to policy-making tended to be specifically within the academic’s research field (*n* = 111 of 188 respondents), rather than partially (*n* = 69 of 188 respondents) or not related (*n* = 8 of 188 respondents). Gender was not significantly associated with these options (χ^2^_2_ = 3.73, P > 0.05; *n* = 161 individual respondents). For participants who had been involved in providing evidence for policy-making in the past, the primary motivations for engaging were for interest (*n* = 138 of 205 respondents) and as a sense of duty as a publicly-funded researcher (*n* = 123 of 205 respondents; see Fig 4). Male participants were more likely than female participants to report that a sense of duty as a motivation for providing evidence (χ^2^_1_ = 7.66, P < 0.01), with no significant gender differences found in the remaining options (χ^2^_1_ < 1.81, P > 0.05 for all; *n* = 175 individual respondents).

### Future provision of evidence by a sub-sample of UK-based research professionals

The EIS aims to create an efficient and effective communication channel between research-producers and research-users in the service of evidence-informed decision-making [26]. The EIS is a potential way of matching academics with Parliamentary staff who need swift access to evidence and knowledge to support their work for politicians and Committees. It may also have wider applications to other groups of people who would gain a benefit from academic advice. The survey tested academic interest in engaging with such a model. Of 285 respondents, 211 stated that they would in principle like to take part in such a rapid response model as the EIS, with a further 69 selecting the ‘Maybe’ option. Having previous policy experience was not significantly related to whether participants selected ‘Yes’ or ‘Maybe’ (GLM: χ^2^_1_ = 0.0084, P > 0.05), but gender did have a marginally significant association with men more likely to select ‘Yes’ (GLM: χ^2^_1_ = 3.94, P < 0.05; *n* = 260 individual respondents). There was no significant difference between disciplines in selecting ‘Yes’ or ‘Maybe’ (GLM: χ^2^_1_ = 0.18, P > 0.05; *n* = 251 individual respondents).

For a rapid response academic-policy model such as the EIS to succeed, research professionals would also need to respond to requests for evidence provision in a short timescale. A combined 278 of 291 respondents stated that they would be prepared to spend time at short notice to respond to research questions in their area of specialism. This was broken down as a quick telephone call / email being the marginally more popular option to provide evidence (*n* = 231 of 291 respondents), relative to a lengthier telephone call / email (*n* = 209 of 291 respondents) and contributing to a policy briefing (*n* = 208 of 291 respondents). Participants were asked to state their likelihood of providing a response to a request for evidence within certain deadlines (see S1 Fig). A quick response was very likely from two days onwards (*n* = 144 of 276 respondents), whereas a lengthier response was only very likely to be provided from one week onwards (*n* = 139 of 279 respondents). Contribution to a policy briefing was very likely from two weeks onwards (*n* = 141 of 276 respondents).

**Fig 5:**
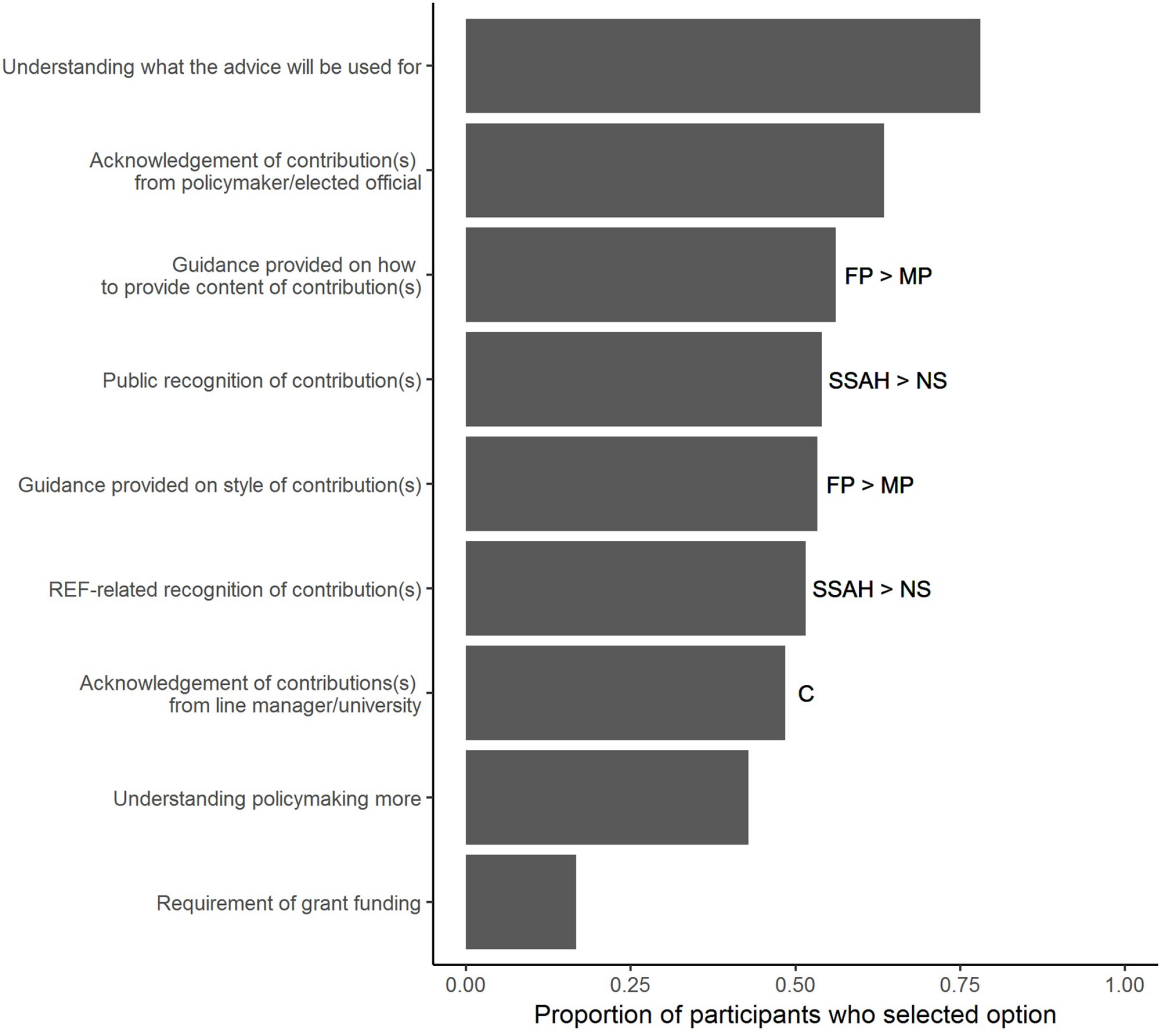
The reported encouragements for contributing to an academic-policy initiative such as the EIS (*n* = 287 individual respondents). Looking at those responses where gender information was also provided, female participants (FP) were significantly more likely to select the two guidance-related options than male participants (MP) (*n* = 267 individual respondents; as indicated by FP > MP). Looking at those responses where career information was also provided, ‘early-career’ were significantly more likely and ‘mid-career’ least likely to select acknowledgement of contributions(s) from line manager/university (see Methods for definitions of career stage; *n* = 210 individual respondents; as indicated by C). Looking at those responses where science discipline information was also provided, ‘social sciences/arts and humanities’ (SSAH) were significantly more likely to select the public and REF-related recognition options (*n* = 255 individual respondents; as indicated by SSAH > NS).

Participants were also asked what would encourage them to contribute to the EIS. The most common selection was to understand what the advice will be used for (224 of 287 respondents; see Fig 5 for full results). Female participants were significantly more likely than male participants to select the options relating to guidance (style: χ^2^_1_ = 12.84, P < 0.001; content: χ^2^_1_ = 15.81, P < 0.001; *n* = 267 individual respondents), with the remaining options not significantly different between the genders (χ^2^_1_ < 2.18, P > 0.05 for all). Participants who were defined as ‘mid-career’ selected acknowledgement of contributions(s) from line manager/university as a form of encouragement most often, with ‘early-career’ selecting this option significantly less (see Methods for definitions of career stage; χ^2^_2_ = 11.30, P < 0.01; *n* = 210 individual respondents). The remaining options were not significantly different between defined career stages (χ^2^_2_ < 3.90, P > 0.05 for all). Respondents from a social sciences/arts and humanities discipline were significantly more likely than natural sciences to select public (χ^2^_1_ = 7.97, P < 0.05) and REF-related (χ^2^_1_ = 8.53, P < 0.05; *n* = 255 individual respondents) recognition as ways to encourage engagement. The remaining options were not significantly different between defined science disciplines (χ^2^_1_ < 5.48, P > 0.05 for all). Two themes emerged from the forty responses that were recorded under ‘Other’: (i) feeling that the participant is making a difference, and (ii) receiving feedback on the use of advice provided (see S2 Appendix).

**Fig 6:**
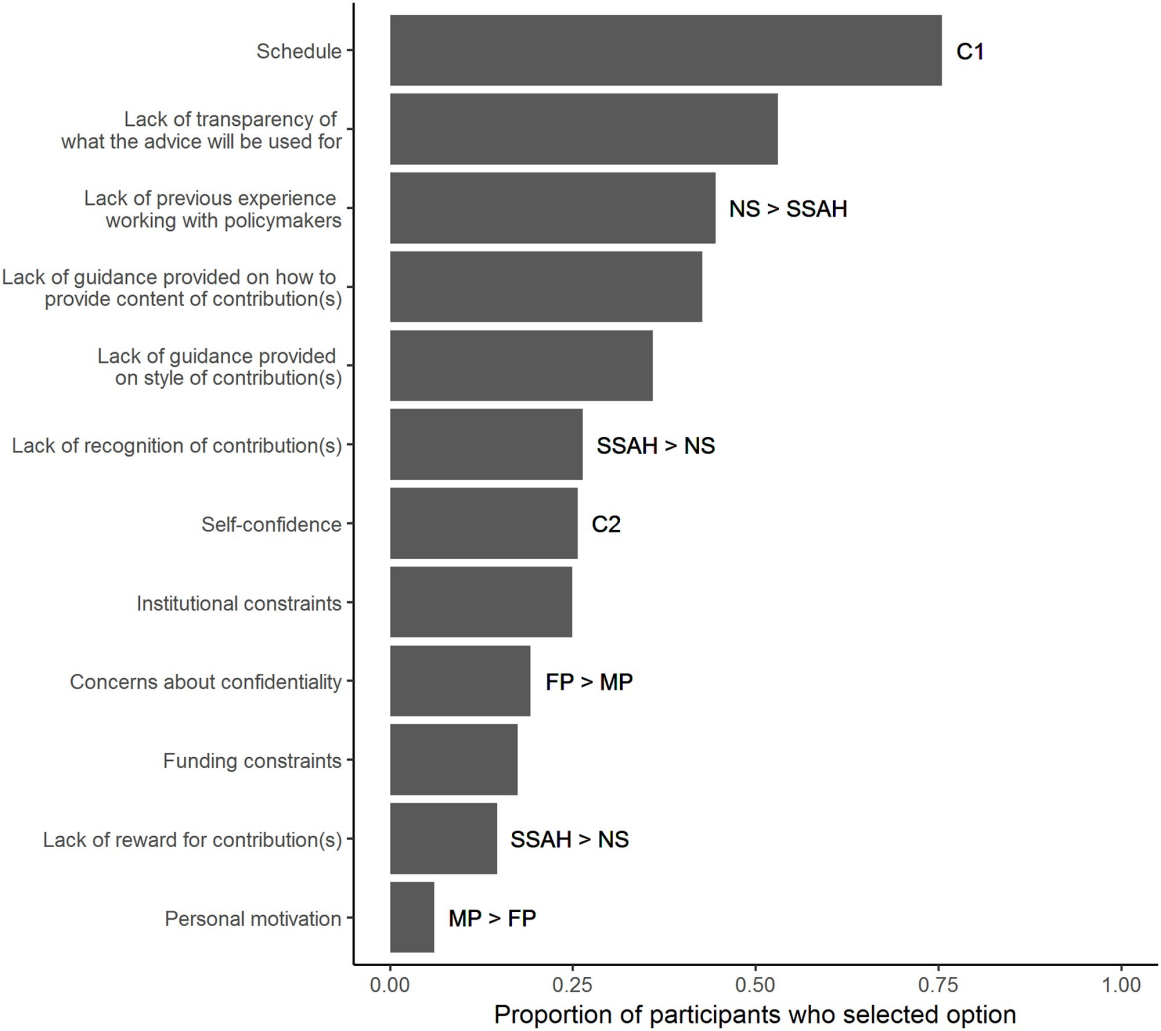
The reported challenges for contributing to an academic-policy initiative such as the EIS (*n* = 281 individual respondents). Looking at those responses where gender information was also provided (*n* = 260 individual respondents), (i) female participants (FP) were significantly more likely to select concerns regarding confidentiality, and (ii) male participants (MP) were significantly more likely to select personal motivation (as indicated by FP > MP and MP > FP respectively). Looking at those responses where career information was also provided, ‘senior-career’ were significantly more likely to select schedule and least likely to select self-confidence than ‘early-career’ or ‘mid-career’ (see Methods for definitions of career stage; *n* = 211 individual respondents; as indicated by C1 and C2 respectively). Looking at those responses where science discipline information was also provided (*n* = 251 individual respondents), ‘social sciences/arts and humanities’ (SSAH) were significantly (i) more likely to select the recognition and reward options, and (ii) less likely to select previous experience, than natural sciences (as indicated by SSAH > NS and NS > SSAH respectively).

The most prominent challenge to contributing to the EIS as identified by the participants is schedule (212 of 281 respondents; see Fig 6 for full results). Female participants were marginally significantly more likely than male participants to select concerns about confidentiality (χ^2^_1_ = 4.06, P < 0.05) as challenges to engaging with a system such as the EIS, while men were significantly more likely than female participants to select personal motivation (χ^2^_1_ = 4.49, P < 0.05; *n* = 260 individual respondents). All other remaining options were not significantly different between the genders (χ^2^_1_ < 3.36, P > 0.05 for all). Self-confidence was selected less often by participants who were defined as ‘senior-career’ (χ^2^_2_ = 9.87, P < 0.01; *n* = 211 individual respondents), whereas schedule was selected less often by ‘early-career’ participants (see Methods for definitions of career stage; χ^2^_2_ = 12.99, P < 0.01). The remaining options were not significantly different between defined career stages (χ^2^_2_ < 3.63, P > 0.05 for all). Respondents from a social sciences/arts and humanities discipline were significantly more likely than natural sciences to select lack of recognition (χ^2^_1_ = 6.47, P < 0.05) and lack of reward (χ^2^_1_ = 5.22, P < 0.05; *n* = 251 individual respondents) for contributions as challenges to engagement. Those with a natural science discipline were more likely than social sciences/arts and humanities to select lack of experience as a challenge to engaging (χ^2^_1_ = 11.55, P < 0.001). The remaining options were not significantly different between defined science disciplines (χ^2^_1_ < 2.37, P > 0.05 for all). Examples specified under the ‘Other’ option (*n* = 21) included reference to the respondents’ view that evidence can be politicised (see S3 Appendix).

### Format of academic-policy initiatives

Respondents indicated preference for research or advice provided to an academic-policy initiative such as the EIS to be publicly available (236 of 285 individual respondents). With regards to anonymity, not knowing the identity of the end-user of the research provided to policy-making would only prevent 21 participants (of 283 respondents) from contributing, and 176 participants (of 284 individual respondents) would prefer to be identifiable themselves to the end-user. Respondents would prefer to be initially contacted about providing evidence for academic-policy initiatives via e-mail (*n* = 269 of 279 respondents) rather than telephone (*n* = 9 of 279 respondents) or an App. interface (*n* = 1 of 279 respondents). 107 of 285 individuals stated that it would be ‘somewhat important’ to have their contributions recognised, followed by the options of ‘important’ (*n* = 93), ‘very important’ (*n* = 57) and ‘not at all important’ (*n* = 28). Gender was independent of this choice (χ^2^_3_ = 2.15, P > 0.05; n = 264 individual respondents).

## Discussion

We used a nationwide online questionnaire to investigate the policy experience of UK-based researchers to better understand the ‘supply-side’ of the academia-policy engagement relationship. Our main findings, for our survey respondents at least, reveal first there are gender-related differences in policy-related experience, as well as motivations and challenges for UK research professionals to contribute to evidence-informed decision-making through initiatives such as the EIS. Second, that understanding what the advice would be used for is a key incentive for academic engagement. Third, that schedule is the main reported barrier to providing research evidence. Fourth, the survey revealed that participants showed strong support for a system such as the proposed EIS that matches expertise with need. Finally, our results indicated that research findings would be provided within short timescales in response to requests for evidence by decision-makers. We discuss the wider implications of these outcomes from our survey respondents for academia-policy engagement initiatives.

Our findings highlight the wider incentives for research professionals to engage with decision-makers. Participants who had previously provided evidence for policy-making reported interest and a sense of duty as publicly funded researchers as key motivators to engaging. This signals a baseline willingness in the academic community to engage with decision-makers, at least within this self-selecting sample. As for encouraging future contributions, participants primarily wanted to understand what the advice would be used for. Two other important key factors in encouraging research professionals to engage with academic-policy initiatives such as the EIS were to have their contributions acknowledged by a policy-maker / elected official, and to have guidance on how to provide content of contribution. Female participants were particularly likely to choose the latter, alongside guidance on style of contribution. Acknowledgement of contribution(s) from a line manager / university was more important for early-career researchers (as defined by this study; see Methods for classification). This may simply be as a result of the academic employment structure, where those defined as early-career are more likely to have supervisors or Principle Investigators rather than be independent researchers. Indeed, acknowledgement from universities might also be perceived to help with career progression at this stage.

Our findings also highlight the wider challenges for research professionals to engage with decision-makers, providing clear guidance on how to improve wider policy engagement with academics. Despite the reported importance for research to be considered during policy-making, only around half of the participants had engaged with a source of material containing evidence that politicians could use. A key challenge for research professionals to contribute is schedule, with participants defined as senior-career researchers (see Methods for classification) more likely to select this option. For REF2014, 20% of impact case studies outlined engagement with the UK Parliament [32]: if universities want to increase this type of engagement, the major barrier of academic workload will require addressing. Corresponding with the top motivator to contribute, a second key challenge was a lack of transparency of what the advice would be used for. Interestingly, lack of previous experience working with policymakers was also reported as a challenge to contributing; however, our results show that a lack of previous policy-related experience would not hinder future participation in academic-policy initiatives such as the EIS.

Combined, these identified challenges and incentives suggest that any academic-policy engagement initiative requires three key features from the academic community’s perspective. First, guidance should be provided for academics tailored to the different opportunities to submit research evidence. Our findings suggest that this may particularly encourage female academics to engage. Providing guidance might also mediate the finding that a lack of previous experience working with policy-makers poses a challenge to contributing, despite the fact that lack of actual experience did not affect reported inclination to participate in an academic-policy initiative such as the EIS. Second, how and why the submitted research evidence will subsequently be used should be made transparent. This may be dealt with at least in part by raising awareness of the differences between policy-related processes, for example policy-making by the Government / Executive or policy scrutiny by Parliament. Third, and related, initiatives should provide clear acknowledgement of the research contribution by academic sources. This last point in particular may have important beneficial implications for REF impact case studies. REF2014 required that there was a direct link from impact case studies to research outputs [8]. Due to the nature of parliamentary scrutiny and policy-making, it is often difficult to pinpoint the influence of one study. Additionally, this requirement to link impact case studies with research outputs potentially excluded impact generated via policy advice [8]. It is possible that future REF and/or KEF iterations may allow for channels involving the provision of policy advice from a wider base of research to be submitted as impact case studies. If academic-policy initiatives are able to provide acknowledgement of research contributions, the cost of preparing impact case studies for submission to REF could conceivably be reduced. In 2014, this was estimated to be £55m and borne by the Higher Education community [8].

The General Election of 2017 delivered the most diverse UK Parliament yet, with a rise in the number of women, LGBT and ethnic minority MPs [33]. However, less is known about the diversity of people who engage with the different parliamentary sites that use research evidence across the UK legislative bodies. Submitting evidence either orally or in writing to a Select Committee is currently one of the key mechanisms for researchers to engage. In 2013–14, only 26.2% of oral evidence was presented by women within the higher education sector to UK Parliament Select Committees [34]. This was marginally higher than the overall gender balance, where 24.6% of oral evidence was given by women in the same study [34]. Similarly for the National Assembly for Wales, it was found that only 27% of external non-elected participants were women in Committee meetings across a twelve-year period [35]. Our results correspond with this low gender diversity, with men more likely to report being asked for general advice and also contributing to a larger number of sources of evidence available to decision-makers. For UK Parliamentary Select Committees, gender diversity data of witnesses has been systematically collected since the publication of a number of recommendations for a more representative and inclusive House of Commons in 2016 [36]. The overall gender diversity of witnesses increased in the 2016–2017 Session to 28.5% women presenting evidence [37]. The UK Parliament is committed to working with a diverse range of people, looking broader than just gender diversity [38]; the devolved administrations also have diversity action plans, see [39–41]. For example, the Universities Programme at the Houses of Parliament recently launched an online survey to identify the barriers for typically under-represented groups to engaging with Parliament for academics [38]. Although our survey could only reliably analyse one facet of diversity—gender—the findings can contribute to this wider intelligence gathering. For academia-policy initiatives to engage female academics, programmes need to address concerns about confidentiality and offer guidance on both style and content of contribution. Understanding and incorporating these gender-related differences in incentives and challenges to engaging with policy-making processes could improve the gender diversity of those who contribute. Expansion of gender diversity in the response base could conceivably increase the quality of evidence by providing broader perspectives and ideologies on research topics [42].

Participants indicated strong support for a system such as the proposed EIS that matches expertise with need. For a rapid response academic-policy model to succeed, research professionals would also need to provide evidence within the short timeframes typical of parliamentary research units and / or decision-makers. This would certainly complement the rapid response parliamentarians indicated they would want to receive information [26]. Our findings suggest that, for our sub-sample at least, UK-based research professionals would be likely to work in a quick turnover capacity, for example providing a quick answer within two days and a lengthier contribution from one week. Future work could test this indication in practice and evaluate the added-value of academic contributions. Additionally, this study only explored the supply-side of a potential academic-policy initiative. Although previous research with UK MPs indicated support for a rapid response system such as the EIS [26], in practice evidence provision is likely to be via existing parliamentary research services as suggested by the parliamentarians themselves. It is therefore important to ascertain the demand by parliamentary research services for academic-policy initiatives such as the EIS. Further research on this particular ‘demand-side’ would be informative for developing effective academic-policy initiatives.

### Limitations

Despite the extent to which the survey was circulated, the uptake was not as high as expected. This could potentially be due to two reasons. First, it may be that only a small number of research professionals received the survey. The survey was primarily disseminated to learned societies and professional services roles (such as Impact Managers, Head of Colleges) with a request to circulate to staff or society members. Instead of facilitating uptake this could potentially have acted as a barrier if the ‘gate-keeper’ decided not to circulate. Linked to this, the dissemination of the survey beyond the initial contact could not be monitored, so the full reach (or lack) of the survey could not be assessed. Second, it may be that only a small number of research professionals are interested in research-policy initiatives. This could conceivably limit the success of research-policy initiatives such as the EIS. However, tailoring research-policy initiatives to include the recommendations from this study may provide the incentives for more research professionals to be actively involved. A second study limitation was the method of delivery. Although online surveys provide a cost-effective method for data gathering, the results are not typically representative of the population as a whole and results should therefore be interpreted with this caveat. For example, not everyone is able to access, use or respond to an online format. Additionally, as the respondents are a self-selecting group the results presented here might only be true of research professionals who are already interested in research-policy initiatives. While this may provide evidence on barriers and challenges of those research professionals with experience or knowledge of engaging (which is valuable data for improving such systems), it may not represent academics in other circumstances. A third study limitation was the lack of participant diversity. The reported demographic information revealed that although gender and age of participants were fairly balanced, diversity in both location of work and ethnic group were low. This may signal that the channels of communication used had biases within them, the phrasing of the survey invite was not perceived as inclusive, or a combination of these or other factors. As the majority of participants reported their place of work as England (outside London), and a majority reported their ethnic group as White, our findings may not be representative for the full diversity of UK-based research professionals. With this in mind, it would be beneficial for future work to invite representatives from minority backgrounds to be a part of the study design to ensure a breadth in participation.

### Conclusion

To conclude, as with our initial consultations with UK parliamentarians [26], we have found a strong appetite within our sub-sample of UK-based research professionals for a system such as the EIS to facilitate connections with decision-makers. Given that many participants had not previously engaged with policy-making yet indicated that it was important for research to be considered, this represents an untapped resource for UK academia-policy initiatives. Key considerations for encouraging diversity and breadth of academic engagement with decision-makers are to; (i) provide tailored guidance, (ii) acknowledge contributions, and (iii) ensure transparency for what the submitted evidence will subsequently be used for. The authors note that the third point may be addressed at least in part by raising the awareness of the different policy-related processes, for example Parliament versus the Executive. Policy-academic evidence-based engagement is a two-way, iterative process with benefits to both sides. An understanding of the motivations of parliamentarians and policy-makers in accessing the best available evidence has been well-documented in the policy literature (e.g. the Executive: [43]; Parliament: [26]). By understanding the motivations and challenges also currently facing research professionals in engaging with such decision-makers, more iterative policy-academic initiatives (like the EIS) could enhance the integration of research evidence with policy and practice across the UK, and even internationally in other similarly democratic political systems.

## Supporting information

S1 Appendix Examples specified under the ‘Other’ option in question 3 of the survey. (DOCX)

S2 Appendix Examples specified under the ‘Other’ option in question 8 of the survey. (DOCX)

S3 Appendix Examples specified under the ‘Other’ option in question 9 of the survey. (DOCX)

S1 Fig Reported likelihoods of providing evidence to certain outputs within specified timeframes. Participants could select a likelihood category for each specified timeframe. The line graphs depict the timeframe in which a response becomes ‘very likely’, as indicated by the solid line in each graph. **A**. A quick response (*n* = 276 individual respondents). **B**. A lengthier response (*n* = 279 individual respondents). **C**. A briefing paper (*n* = 276 individual respondents).
(TIFF)

S1 Table Questions included in the survey distributed by Qualtrics. (DOCX)
