# Supplementary material for: Supporting evidence-informed policy and scrutiny: A consultation of UK research professionals
Source: PLoS One. 2019 Mar 26;14(3):e0214136. doi: 10.1371/journal.pone.0214136 (PMC6435130; doi:10.1371/journal.pone.0214136)
Supplement: S1 Appendix — (DOCX) [file pone.0214136.s001.docx]

# S1 Appendix

Examples specified under the ‘Other’ option (*n* = 12; question 3 in S1 Table) included several references to both government/ and parliamentary process, such as: *participated in a House of Commons roundtable discussion*; *membership of Defra Evidence groups*; *invited to present at the Speakers House in Westminster*.
