# Supplementary material for: Supporting evidence-informed policy and scrutiny: A consultation of UK research professionals
Source: PLoS One. 2019 Mar 26;14(3):e0214136. doi: 10.1371/journal.pone.0214136 (PMC6435130; doi:10.1371/journal.pone.0214136)
Supplement: S2 Appendix — (DOCX) [file pone.0214136.s002.docx]

# S2 Appendix

Of the information provided in ‘Other’ (*n* = 40; question 8 in S1 Table), several participants responded within the broader theme of feeling that they are making a difference. For example:

*“Actually being listened to and being able to make the world a better place”*

*“Responsibility as a publicly funded scientist to support societal progress”*

*“Sense of knowing that I had a useful contribution = that could make a difference”*

*“Seeing it put into practice for greater good*.*”*

A second theme that emerged was receiving feedback on the use of advice provided, for example:

*“Being informed of the outcome and feedback on usefulness”*

*“That contribution leads to evidence based policy making”*

*“Confidence regarding how the advice will be used”*

*“Feedback on how the advice was actually used / what the impact was”*

*“Likelihood of effective use of advice”*
