# Supplementary material for: Supporting evidence-informed policy and scrutiny: A consultation of UK research professionals
Source: PLoS One. 2019 Mar 26;14(3):e0214136. doi: 10.1371/journal.pone.0214136 (PMC6435130; doi:10.1371/journal.pone.0214136)
Supplement: S3 Appendix — (DOCX) [file pone.0214136.s003.docx]

# S3 Appendix

Examples specified under the ‘Other’ option (*n* = 21; question 9 in S1 Table) included reference to the respondents’ view that evidence can be politicised:

*“Policymakers do not want to hear about evidence that does not confirm their view of the world or political position”*

*“End use of advice unacceptable”*

*“Very likely that in my fields […] my contribution would be ignored. But it is important to get some research into the public record nevertheless”*

*“Having done it - recognition that comments only 'used' if seen as politically/ideologically relevant*.”
