# Supplementary figures and images for: Supporting evidence-informed policy and scrutiny: A consultation of UK research professionals
Source: PLoS One. 2019 Mar 26;14(3):e0214136. doi: 10.1371/journal.pone.0214136 (PMC6435130; doi:10.1371/journal.pone.0214136)

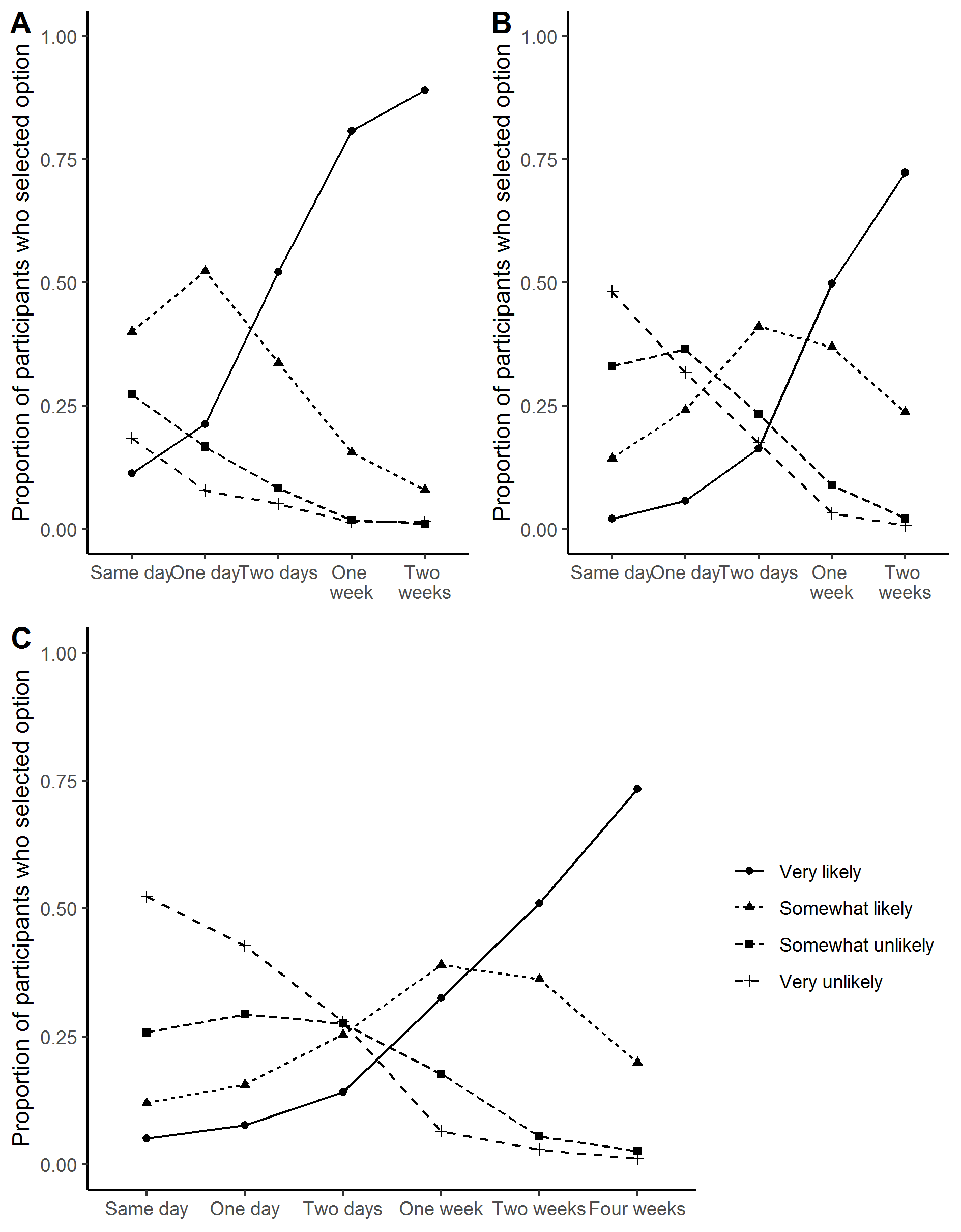

Supplement: S1 Fig — Participants could select a likelihood category for each specified timeframe. The line graphs depict the timeframe in which a response becomes ‘very likely’, as indicated by the solid line in each graph. A. A quick response (n = 276 individual respondents). B. A lengthier response (n = 279 individual respondents). C. A briefing paper (n = 276 individual respondents). (TIFF) [file pone.0214136.s004.tiff]
