# Supplementary material for: Supporting evidence-informed policy and scrutiny: A consultation of UK research professionals
Source: PLoS One. 2019 Mar 26;14(3):e0214136. doi: 10.1371/journal.pone.0214136 (PMC6435130; doi:10.1371/journal.pone.0214136)
Supplement: S1 Table — (DOCX) [file pone.0214136.s005.docx]

| **By completing this anonymous survey, you are providing consent for participation in this survey** | | | | | | | | | |
| --- | --- | --- | --- | --- | --- | --- | --- | --- | --- |
| I agree | | | | | | | | | |
| I do not agree | | | | | | | | | |
| **Section 1. Experience of evidence-based policymaking.** | | | | | | | | | |
| **Q1. Academic research is frequently used to support policymaking. Do you feel that** | | | | | | | | | |
| It is important for research to be considered during policymaking | | | | | | | | | |
| It is not important for research to be considered during policymaking | | | | | | | | | |
| Unsure | | | | | | | | | |
| **Q2. The following are sources of material containing evidence that politicians could use. Have you ever (i) heard of, (ii) been approached by those involved but decided not to contribute, or (iii) contributed to any of the following?** | | | | | | | | | |
| *SOURCE* | | *HEARD OF ONLY* | | *APPROACHED BY, BUT NOT CONTRIBUTED* | | *CONTRIBUTED TO* | | *NOT HEARD OF* | |
| Reviews of evidence from Government | |  | |  | |  | |  | |
| Government papers | |  | |  | |  | |  | |
| Reports from Think Tanks | |  | |  | |  | |  | |
| House of Commons Select Committee reports | |  | |  | |  | |  | |
| House of Lords Select Committee reports | |  | |  | |  | |  | |
| House of Commons Library | |  | |  | |  | |  | |
| National Assembly for Wales Research Service | |  | |  | |  | |  | |
| Scottish Parliament Information Centre (SPICe) | |  | |  | |  | |  | |
| Northern Ireland Research and Information Service (RaISe) | |  | |  | |  | |  | |
| Parliamentary Office of Science and Technology | |  | |  | |  | |  | |
| Policy institute/department associate with the University | |  | |  | |  | |  | |
| Journalists for printed/online articles, e.g. commenting on media reports about government policy | |  | |  | |  | |  | |
| **Q3. What was the nature of these interactions? Please select all that apply.** | | | | | | | | | |
| Talk about own research project(s) | | | | | | | | | |
| Provide general advice on research topic | | | | | | | | | |
| Public engagement | | | | | | | | | |
| Part of a research project specifically involving contribution to policymaking | | | | | | | | | |
| Other (please specify) | | | | | | | | | |
| I did not have any interactions | | | | | | | | | |
| **Q4. If you have engaged with any external groups to contribute to policymaking, was this:** | | | | | | | | | |
| Specifically in my research field | | | | | | | | | |
| Partially in my research field | | | | | | | | | |
| No, not related to my field but I provided advice anyway | | | | | | | | | |
| Not applicable | | | | | | | | | |
| **Q5. If you have been involved in providing evidence for policymaking in the past, what motivated you to do so?** | | | | | | | | | |
| Prestige | | | | | | | | | |
| REF purposes | | | | | | | | | |
| Interest | | | | | | | | | |
| Pressure | | | | | | | | | |
| Part of my job e.g. working at evidence-policy interface | | | | | | | | | |
| Sense of duty as a publicly-funded researcher | | | | | | | | | |
| Curiosity | | | | | | | | | |
| **Section 2. The EIS and your potential contribution.**  *The EIS is based on the idea of academics giving some of their time to respond to questions/collate evidence for more informed evidence-based policymaking.* | | | | | | | | | |
| **Q6a. Would you be prepared to spend time at short notice to respond to research questions in your area of specialism? Please tick all that apply.** | | | | | | | | | |
| Yes – prepared to contribute via a quick telephone call / email | | | | | | | | | |
| Yes – prepared to contribute via a lengthier telephone call / email | | | | | | | | | |
| Yes – would consider contributing to a policy briefing | | | | | | | | | |
| No | | | | | | | | | |
| Unsure | | | | | | | | | |
| **Q6b. Please indicate your likeliness of providing a quick response to requests with the following deadlines.** | | | | | | | | | |
| *OPTION* | *VERY LIKELY* | | *SOMEWHAT LIKELY* | | *SOMEWHAT UNLIKELY* | | *VERY UNLIKELY* | | *UNSURE* |
| Same day |  | |  | |  | |  | |  |
| One day |  | |  | |  | |  | |  |
| Two days |  | |  | |  | |  | |  |
| 1 week |  | |  | |  | |  | |  |
| 2 weeks |  | |  | |  | |  | |  |
| **Q6c. Please indicate your likeliness of providing a lengthier response to requests with the following deadlines** | | | | | | | | | |
| *OPTION* | *VERY LIKELY* | | *SOMEWHAT LIKELY* | | *SOMEWHAT UNLIKELY* | | *VERY UNLIKELY* | | *UNSURE* |
| Same day |  | |  | |  | |  | |  |
| One day |  | |  | |  | |  | |  |
| Two days |  | |  | |  | |  | |  |
| 1 week |  | |  | |  | |  | |  |
| 2 weeks |  | |  | |  | |  | |  |
| **Q6d. Please indicate your likeliness of responding to a request to contribute to a policy briefing with the following deadlines** | | | | | | | | | |
| *OPTION* | *VERY LIKELY* | | *SOMEWHAT LIKELY* | | *SOMEWHAT UNLIKELY* | | *VERY UNLIKELY* | | *UNSURE* |
| Same day |  | |  | |  | |  | |  |
| One day |  | |  | |  | |  | |  |
| Two days |  | |  | |  | |  | |  |
| 1 week |  | |  | |  | |  | |  |
| 2 weeks |  | |  | |  | |  | |  |
| 4 weeks |  | |  | |  | |  | |  |
| **Q7. How frequently would you be willing to contribute to the EIS?** | | | | | | | | | |
| 1-2 times a year | | | | | | | | | |
| 3-5 times a year | | | | | | | | | |
| 5+ times a year | | | | | | | | | |
| It depends on the nature of the enquiries | | | | | | | | | |
| **Q8. What would encourage you to contribute to the EIS? Please tick all that apply.** | | | | | | | | | |
| Public recognition of contribution(s) | | | | | | | | | |
| REF-related recognition of contribution(s) | | | | | | | | | |
| Acknowledgement of contribution(s) from policymaker/elected official | | | | | | | | | |
| Acknowledgement of contributions(s) from line manager/university | | | | | | | | | |
| Guidance provided on how to provide content of contribution(s) | | | | | | | | | |
| Guidance provided on style of contribution(s) | | | | | | | | | |
| Understanding what the advice will be used for, e.g. select committee, debate, published report | | | | | | | | | |
| Understanding policymaking more | | | | | | | | | |
| Requirement of grant funding | | | | | | | | | |
| Other (please specify) | | | | | | | | | |
| **Q9. Why might you find it a challenge to contribute to the EIS? Please tick all that apply.** | | | | | | | | | |
| Schedule | | | | | | | | | |
| Self-confidence | | | | | | | | | |
| Lack of previous experience working with policymakers | | | | | | | | | |
| Lack of recognition of contribution(s) | | | | | | | | | |
| Lack of reward for contribution(s) | | | | | | | | | |
| Institutional constraints | | | | | | | | | |
| Funding constraints | | | | | | | | | |
| Personal motivation | | | | | | | | | |
| Lack of guidance provided on how to provide content of contribution(s) | | | | | | | | | |
| Lack of guidance provided on style of contribution(s) | | | | | | | | | |
| Lack of transparency of what the advice will be used for, e.g. select committee, debate, published report | | | | | | | | | |
| Concerns about confidentiality | | | | | | | | | |
| Other (please specify) | | | | | | | | | |
| **Q10. How important would it be for you personally to have any contribution you made to the EIS recognised?** | | | | | | | | | |
| Very important | | | | | | | | | |
| Important | | | | | | | | | |
| Somewhat important | | | | | | | | | |
| Not at all important | | | | | | | | | |
| **Q11. Do you think the research or advice provided by the EIS should be published i.e. publically available?** | | | | | | | | | |
| Yes | | | | | | | | | |
| No | | | | | | | | | |
| This is not important to me | | | | | | | | | |
| **Q12a. Some users of the EIS, such as the House of Commons Library who provide a confidential research service to MPs, may require the end-user (for example, an MP) of any research request to be anonymous. Would you be happy to contribute in these circumstances?** | | | | | | | | | |
| Yes | | | | | | | | | |
| No | | | | | | | | | |
| Unsure | | | | | | | | | |
| **Q12b. As an academic contributing to the EIS, would you prefer** | | | | | | | | | |
| To be anonymous to the end-user (e.g. MP) | | | | | | | | | |
| To be identifiable to the end-user (e.g. MP) | | | | | | | | | |
| Unsure | | | | | | | | | |
| **Q13. Would you in principle like to take part in the EIS?** | | | | | | | | | |
| Yes | | | | | | | | | |
| No | | | | | | | | | |
| Possibly | | | | | | | | | |
| **Q14. How would you prefer to be contacted about contributing to the EIS?** | | | | | | | | | |
| Telephone | | | | | | | | | |
| Email | | | | | | | | | |
| App | | | | | | | | | |
| Other (please specify) | | | | | | | | | |
| **Q15. Who do you think should contribute to the EIS? Please tick all that apply.** | | | | | | | | | |
| All levels of academic | | | | | | | | | |
| MSc researchers | | | | | | | | | |
| Research assistants | | | | | | | | | |
| Research technicians | | | | | | | | | |
| PhD researchers | | | | | | | | | |
| Post-doc levels | | | | | | | | | |
| Teaching Fellows | | | | | | | | | |
| Research Fellows | | | | | | | | | |
| Lecturers | | | | | | | | | |
| Senior Lecturers or Readers | | | | | | | | | |
| Associate Professors | | | | | | | | | |
| Professors | | | | | | | | | |
| Other (please specify) | | | | | | | | | |
| **Q16. Please provide any additional comments you have about the EIS:** | | | | | | | | | |
| *[Free text box]* | | | | | | | | | |
| **Section 3. Finally, we would be grateful if you could provide us with the following demographic information.** | | | | | | | | | |
| **Q17. What is your gender identity?** | | | | | | | | | |
| Female | | | | | | | | | |
| Male | | | | | | | | | |
| Other | | | | | | | | | |
| Prefer not to disclose | | | | | | | | | |
| **Q18. Please select the age group to which you belong.** | | | | | | | | | |
| 18-25 | | | | | | | | | |
| 26-35 | | | | | | | | | |
| 36-45 | | | | | | | | | |
| 46-55 | | | | | | | | | |
| 56-65 | | | | | | | | | |
| 66+ | | | | | | | | | |
| Prefer not to disclose | | | | | | | | | |
| **Q19. Please specify your ethnic group.** | | | | | | | | | |
| White (British, Irish or other) | | | | | | | | | |
| Asian or Asian British | | | | | | | | | |
| Black or Black British | | | | | | | | | |
| Other (please specify) | | | | | | | | | |
| Prefer not to disclose | | | | | | | | | |
| **Q20. Please tell us your academic discipline.** | | | | | | | | | |
| **Q21. Please tell us your academic institution.** | | | | | | | | | |
| **Q22. What is your current position?** | | | | | | | | | |
| MSc researcher | | | | | | | | | |
| Research assistant | | | | | | | | | |
| Research technician | | | | | | | | | |
| PhD researcher | | | | | | | | | |
| Post-doc position | | | | | | | | | |
| Teaching Fellow | | | | | | | | | |
| Research Fellow | | | | | | | | | |
| Lecturer | | | | | | | | | |
| Senior Lecturer or Reader | | | | | | | | | |
| Associate Professor | | | | | | | | | |
| Professor | | | | | | | | | |
| Other (please specify) | | | | | | | | | |
